# Bifunctional Lipocalin Ameliorates Murine Immune Complex-induced Acute Lung Injury[Fn FN1]

**DOI:** 10.1074/jbc.M112.420331

**Published:** 2013-04-26

**Authors:** Pietro Roversi, Bernhard Ryffel, Dieudonnée Togbe, Isabelle Maillet, Mauro Teixeira, Nurfilza Ahmat, Guido C. Paesen, Olga Lissina, Wilhelm Boland, Kerstin Ploss, Joseph J. E. Caesar, Susanne Leonhartsberger, Susan M. Lea, Miles A. Nunn

**Affiliations:** From the ‡ Sir William Dunn School of Pathology, University of Oxford, Oxford OX1 3RE, United Kingdom,; the § Molecular Immunology and Embryology, UMR7355, Orleans University-CNRS, 3b rue de la Ferollerie, F-45071 Orleans, France,; the ¶ Institute of Infectious Disease and Molecular Medicine (IIDMM), University of Cape Town, Rondebosch 7701, Cape Town, South Africa,; the ‖ ArtImmune SAS, F-45071 Orleans, France,; the ** Centre for Ecology and Hydrology, Maclean Building, Wallingford OX10 8BB, F-45071 United Kingdom,; the ‡‡ Department of Bioorganic Chemistry, Max-Planck-Institut für Chemische Ökologie, Hans-Knöll-Strasse 8, D-07745 Jena, Germany, and; §§ Wacker Biotech GmbH, Hans-Knöll- Strasse 3, 07745 Jena, Germany

**Keywords:** Complement, Immunotherapy, Inflammation, Leukotriene, Lung Injury, Parasite, Acute Lung Injury, Immune Complex

## Abstract

Molecules that simultaneously inhibit independent or co-dependent proinflammatory pathways may have advantages over conventional monotherapeutics. OmCI is a bifunctional protein derived from blood-feeding ticks that specifically prevents complement (C)-mediated C5 activation and also sequesters leukotriene B4 (LTB_4_) within an internal binding pocket. Here, we examined the effect of LTB_4_ binding on OmCI structure and function and investigated the relative importance of C-mediated C5 activation and LTB_4_ in a mouse model of immune complex-induced acute lung injury (IC-ALI). We describe two crystal structures of bacterially expressed OmCI: one binding a C16 fatty acid and the other binding LTB_4_ (C20). We show that the C5 and LTB_4_ binding activities of the molecule are independent of each other and that OmCI is a potent inhibitor of experimental IC-ALI, equally dependent on both C5 inhibition and LTB_4_ binding for full activity. The data highlight the importance of LTB_4_ in IC-ALI and activation of C5 by the complement pathway C5 convertase rather than by non-C proteases. The findings suggest that dual inhibition of C5 and LTB_4_ may be useful for treatment of human immune complex-dependent diseases.

## Teaser

**Background:** OmCI is an ectoparasite-derived anti-inflammatory protein that binds LTB_4_ and prevents complement C5 activation.

**Results:** The C5 and LTB_4_ binding activities of OmCI are functionally and structurally independent, and OmCI potently inhibits immune complex-induced acute lung injury (IC-ALI).

**Conclusion:** LTB_4_ and C5 activation by complement contribute equally to the pathology of IC-ALI.

**Significance:** Dual inhibition of these mediators should be considered for treatment of IC-dependent diseases.

## Introduction

The immune response depends upon coordinated release and orchestration of diverse mediators. Molecules that simultaneously inhibit mediators that have independent or co-dependent proinflammatory effects may have advantages over conventional therapeutics such as mAb, which normally target single components of the immune system. Possible advantages of targeting more than one pathway include higher specificity, lower effective dose (1), and the ability to counteract compensation mechanisms within biological networks (2, 3).

The 17-kDa lipocalin *Ornithodoros moubata* complement inhibitor OmCI (4), originally isolated from an ectoparasitic tick (Acari), is a bifunctional protein that may have such therapeutic advantages. It captures the proinflammatory eicosanoid leukotriene B4 (LTB_4_)^8^ within an internal binding cavity (data presented herein) and also prevents complement (C)-mediated activation of C component 5 (C5) in a wide range of mammalian species including humans (5). By binding directly to C5 in the vicinity of the C5-C345C domain OmCI prevents cleavage of C5 by the C5 complement convertases, thereby preventing release of anaphylatoxin C5a and formation of the terminal 5b-9 C complex (TCC) (6–8). OmCI therefore circumvents the effects of the TCC, and the cell surface G protein-coupled receptors activated by LTB_4_ (BLT1 and BLT2 receptors) and C5a (C5aR). OmCI may also prevent activation of the non-G protein-coupled C5L2 receptor for C5a. The function, and even the cellular location, of C5L2 is subject to ongoing debate with both pro- and anti-inflammatory activities described (9).

The established downstream effects of the TCC and C5aR, BLT1, and BLT2 signaling are numerous and interlinked. LTB_4_, derived like all eicosanoids from arachidonic acid (AA), and activated C5 both have rapid and vital roles in the initiation and coordination of the early inflammatory and adaptive immune responses (reviewed in Refs. 10–14). Among other effects, TCC formation on self-cells induces release of inflammatory mediators including IL-6, synthesis of AA derivatives, transendothelial migration of polymorphonuclear leukocytes, and production of active oxygen metabolites (reviewed in Ref. 15). Both C5a and LTB_4_ rapidly recruit and activate granulocytes (in particular neutrophils) and monocytes and trigger oxidative burst and degranulation (14–17), resulting in the release of numerous preformed proinflammatory and vasoactive mediators (histamine, serotonin, tryptase, and defensins) and proteases that can generate C5a independently of C (18, 19). These actions stimulate the production of proinflammatory cytokines (IL-1, IL-2, IL-6, IL-8, and TNFα), chemokines (eotaxin, RANTES, and MIP2), growth factor (TGFβ), LTB_4_, and other eicosanoids that augment and prolong tissue inflammation (20, 21). C5a alone induces vasodilation and smooth muscle cell contraction, whereas both C5a and LTB_4_ increase microvascular permeability (10, 13). LTB_4_ amplifies the neutrophil chemotactic effect of C5a in inflammatory processes, and conversely, the release of AA and synthesis of LTB_4_ can be stimulated by both TCC and C5a (10, 15, 22–24).

Marketed therapies target C5 or leukotrienes. C is the focus of much recent drug research and development (10, 25), and a humanized anti-C5 mAb (eculizumab) successfully treats nocturnal paroxysmal hemoglobinuria (26). Eculizumab is in clinical trials for the treatment of a variety of other pathologies including atypical hemolytic uremic syndrome and kidney transplant rejection (60, 61). Therapies targeting leukotrienes are more advanced (27). Glucocorticoids inhibit the release of AA (28). Other drugs approved for treatment of chronic asthma target leukotrienes directly by inhibiting the 5-LOX enzyme required for LTB_4_ and cysteinyl leukotriene (cysLT) synthesis (zileuton (29)) or antagonize the high affinity receptor CysLT_1_R that mediates most of the effects of the cysLTs (zafirlukast and montelukast (30)). No drug that acts specifically on LTB_4_ or its receptors has yet reached the market, but many are in development (31). The effect of the combined inhibition of C5 and LTB_4_ has not been reported.

Given the direct and indirect interactions between C and LTB_4_ and the efficacy of marketed drugs targeted at C5 and LTs, we hypothesized that combined inhibition of these components might potently inhibit inflammation. Here, we examined the effect of LTB_4_ binding on structure and function of OmCI and investigated the relative importance of C-mediated C5 activation and LTB_4_ in a mouse model (9, 32–37) of immune complex-induced acute lung injury (IC-ALI).

## EXPERIMENTAL PROCEDURES

### 

#### 

##### Materials

[5,6,8,9,11,12,14,15-^3^H(*n*)]LTB4 (lot 3589956; total activity 5 μCi or 185 kBq; specific activity 190 Ci/mmol) was from PerkinElmer Life Sciences. Fatty acid ELISA kits were from Assay Designs, Inc. The C5a assay kit was from DRG Diagnostica (catalog No. RE59292). All surface plasmon resonance (SPR) reagents were from Biacore (Uppsala, Sweden). Sheep RBC were from Tissue Culture Services. Hemolysin, pooled normal human sera, GVB^2+^, and human thrombin (catalog No. T6884) were from Sigma. Guinea pig serum and the control proteins RaHBP2 (*Rhipicephalus appendiculatus* histamine-binding protein 2) and OmCLI (*O. moubata* cysteinyl leukotriene inhibitor) were derived in-house. Human C5 (hC5) was purchased from Calbiochem (catalog No. 204888). The 5-lipoxygenase-activating enzyme inhibitor MK886 was purchased from Tocris Bioscience (Bristol, UK), and LTB_4_, leukotriene C_4_ (LTC_4_), 12(*S*)-hydroxyeicosatetraenoic acid (12(*S*)-HETE), and AA were bought from Cayman Chemical (Ann Arbor, MI) and Biomol International (Exeter, UK).

##### Recombinant OmCI

Soluble bacterially expressed OmCI (bOmCI) was manufactured using the proprietary technology of Wacker GmbH (38). *Escherichia coli* strain WCM105 containing a plasmid for expression and secretion of OmCI was cultivated in a 30-liter bioreactor (type Biostat C-20; Sartorius AG, Melsungen, Germany). Fermentation was performed at 30 °C in a mineral salt medium including trace elements, yeast extract, and phytone at pH 7.0 under aerobic conditions with a continuous glucose feed. Prior to inducing expression with IPTG, the temperature was dropped to 20 °C. Approximately 36 h after induction, the supernatant was harvested by centrifugation (CEPA Z41, Padberg GmbH, Germany) and filtered using a 0.45-μm membrane filter (Sartopure PP2) and then a 0.2-μm membrane filter (Sartopore 2HF). OmCI was purified from the supernatant as described (7) with a final additional chromatographic step in which the active fractions were adjusted to 2 m ammonium sulfate and then loaded onto phenyl-Sepharose (GE Healthcare) and eluted using a 2–0.5 m ammonium sulfate gradient. Active fractions were dialyzed against PBS, analyzed by denaturing SDS-PAGE, and concentrated to 8.4 mg/ml by ultrafiltration. A predicted *A*_280_^0.1%, 1 cm^ of 1.3 was used to estimate the protein concentration. The pure protein (94% by RP-HPLC (supplemental Fig. S1) had a final yield of ∼0.4 g/liter of fermentation broth. The identity and activity of the protein were confirmed by ESI-Q-TOF MS and by inhibition of a classical complement pathway assay used in accordance with the manufacturer's instructions (CH50 Eq ELISA from Quidel). The experimentally estimated *M*_r_ (16,779.0 g/mol) matched the calculated *M*_r_ of 16,779.5 g/mol.

##### GC-MS Analysis of Fatty Acids from Recombinant bOmCI

Extraction and analysis of fatty acids from the recombinant protein was performed as described (6). Mass spectrometric analysis was performed on a Trace MS (Thermo Finnigan, D-63329, Egelsbach, Germany) equipped with fused silica Alltech EC5 (D-82008, Unterhaching, Germany) capillary (15 m × 0.25 mm, 0.25 μm) using helium at 1.5 ml min^−1^ as the carrier gas.

##### Preparation of LTB_4_-saturated OmCI (bOmCI-LTB_4_)

bOmCI (4.5 mg) was incubated with 2 ml of LTB_4_ (50 ng/μl stock in ultrapure ethanol) in 39 ml of PBS, pH 7.2, at room temperature with shaking for 10 min. This 1:1.1 molar ratio mixture was concentrated to 200 μl in a 5-kDa cut-off Vivaspin ultrafiltration device (Sartorious). The retentate was washed with 30 ml of PBS, pH 7.2, and reconcentrated to 200 μl. In parallel, the same amount (4.5 mg) of bOmCI was incubated with 2 ml of ultrapure ethanol in 39 ml of PBS and then washed and concentrated as described.

##### Measurement of Eicosanoid Binding by Enzyme Immunoassay

Correlate-EIA^TM^ kits (Assay Designs, Inc.) for solution measurement of LTB_4_, thromboxane B_2_ (TXB_2_), and cysLT were used. To examine direct binding, 0.1–30 μg of pure OmCI, OmCI saturated with LTB_4_, RaHBP2 (histamine binding), and OmCLI (cysteinyl leukotriene binding) were incubated with 150 μl of the eicosanoids conjugated to alkaline phosphatase (AP). After 20 min of shaking (500 rpm) at room temperature, free eicosanoid-AP was detected by mixing the lipocalin-eicosanoid solution with anti-eicosanoid-specific polyclonal rabbit Ab and adding the solution to a plate coated with goat anti-rabbit Ab. After a 2-h incubation, the plate was washed in accordance with the manufacturer's instructions; at this stage the tick lipocalins including those bound to eicosanoid-AP were washed away. Anti-eicosanoid Ab bound to AP-labeled fatty acids was detected by addition of substrate. The results for each lipocalin were compared with PBS controls.

##### UV Absorption Spectroscopy of bOmCI Bound to LTB_4_

bOmCI and LTB_4_ in ethanol were mixed in PBS at molar ratios of 0:1, 0.03:1, 0.06:1, 0.0125:1. 0.25:1, 0.5:1, 1:1, 2:1, 4:1, and 8:1 and incubated for 5 min at room temperature. UV absorption spectra (200–400 nm) were recorded using a Nanodrop ND-1000 spectrophotometer. The average spectrum (five replicates) for each protein dilution with ultrapure ethanol but without LTB_4_ was subtracted from the average spectrum for each protein dilution in the presence of LTB_4._ The same assay was undertaken using a 4:1 molar excess of RaHBP2, OmCLI, and OmCI saturated with LTB_4_. Spectroscopy of LTB_4_ with and without OmCI was also undertaken in the presence of a large molar excess of AA (60×) or 12(*S*)-HETE (40×). The average spectrum (five replicates) for each protein dilution with AA or 12(*S*)-HETE but without LTB_4_ was subtracted from the average spectrum for each protein dilution in the presence of LTB_4_ mixed with excess AA or 12(*S*)-HETE.

##### bOmCI Crystallization, Data Collection, and Structure Determination

bOmCI and bOmCI-LTB_4_ crystals were grown by vapor diffusion in 400 nl of sitting drops at 20 °C. The crystallization drops were obtained by mixing 0.2 μl of protein solution with 0.2 μl of the crystallization screen and were equilibrated against 100 μl of mother liquor.

For bOmCI, screens were set up using a TECAN robot (Tecan Group Ltd.). Initial crystallization screens using the molecular dimensions structure screens were set up with bOmCI at 65 mg/ml. Initial crystals grew in conditions 23, 33, and 37 of MD Structure Screen 1; the best crystals grew after setting up repeats of condition 33 (30% PEG 4000, 0.2 m magnesium chloride, and 0.1 m Tris, pH 8.5).

For bOmCI-LTB_4_, bOmCI loaded with LTB_4_ (see “Experimental Procedures”) was concentrated to 25 mg/ml in Tris-HCl, pH 7, 30 mm NaCl. Screens were set up using an OryxNano robot (Douglas Instruments Ltd., UK). Initial crystallization screens using Hampton Research crystal screens were set up with a bOmCI-LTB_4_ mix in 1:1 molar ratios (see above for concentration). Initial crystals grew in conditions 14 and 22 of the Hampton Research Crystal Screen HT; better crystals were grown after screening around condition 22 (21% PEG 4000, 0.2 m sodium acetate, 0.1 m Tris, pH 8.4).

For bOmCI, a 1.9 Å x-ray diffraction data set was collected from a crystal (orthorhombic P2_1_2_1_2_1_, *a* = 44.4 Å, *b* = 51.9 Å, *c* = 68.40 Å, 1 molecule/asymmetric unit) on beamline ID14-2 at the ESRF. For bOmCI-LTB_4_ a 1.9 Å x-ray diffraction data set was collected from a crystal (monoclinic P2_1_, *a* = 41.76 Å, *b* = 112.81 Å, *c* = 62.40 Å, β = 101.89 degrees, 4 molecules/asymmetric unit) on beamline BM14 at the ESRF. All x-ray data integration and scaling were done using the computer programs Mosflm (39) and Scala (40). Supplemental Table S1 shows the crystallographic data collection and processing statistics. Both structures were phased by molecular replacement with the computer program CCP4-Phaser, built in Coot (41), and refined in Buster-TNT (42) using alternating cycles of full B refinement and TLS refinement, with one TLS body per chain. Supplemental Table S2 contains the final crystallographic refinement data and statistics. The yeast-expressed yOmCI coordinates (PDB accession code 2CM4) were used as a search model for bOmCI. The model for the bound palmitoleic acid was built in the residual electron density once the model for the protein had been completed. For bOmCI-LTB_4_, the search model was the bOmCI structure stripped of the ligand. The model for the bound LTB_4_ was built in the residual electron density once the model for the protein had been completed. The initial stages of refinement implemented non-crystallographic symmetry restraints, which were released in the final cycles. The structures were deposited in the Protein Data Bank and have accession codes 3ZUI and 3ZUO.

##### Surface Plasmon Resonance

Measurements were conducted on a Biacore 3000 instrument. 600–1000 resonance units of bOmCI or 200–300 resonance units of bOmCI-LTB_4_ (which coupled less efficiently than bOmCI) were immobilized by amine coupling at pH 4.5 to separate CM5 sensor chips as recommended by Biacore. A control channel was mock coupled with buffer. All experiments were performed at 25 °C in 10 mm HEPES, pH 7.4, 150 mm NaCl, 3 mm EDTA, and 0.005% surfactant P20 with various concentrations of hC5 with and without 10 nm LTB_4_. Analyte solutions were passed through the flow cells sequentially at a flow rate of 40 μl/min. The chip surface was regenerated using 10 mm glycine, pH 1.5, between each injection. Kinetic parameters were evaluated in accordance with the manufacturer's instructions.

##### Classical Hemolytic Assay

Sensitized sheep erythrocytes were prepared in GVB^2+^ buffer. Assays were carried out using 50 μl of 1:320 GVB^2+^ diluted guinea pig serum and 50 μl of 2 × 10^8^ activated erythrocyte cells ml^−1^. This was the maximum serum dilution (1:640 final) that gave ∼100% lysis without inhibitor after 30 min of incubation. The GVB^2+^ buffer was supplemented with a 2-fold molar excess of LTB_4_ to bOmCI. bOmCI or RaHBP2 control protein diluted in PBS was added last (5 μl each), and reactions were incubated at 37 °C for 30 min. Reactions were centrifuged (12000 × *g* for 5 s) and hemolysis measured at 412 nm. Percent lysis was calculated using the absorbance value for 100% cell lysis caused by adding 50 μl of water to 50 μl of erythrocyte cells.

##### Measurement of LTB_4_ Binding Using Radioligand

A 1:2 molar ratio of bOmCI and hC5 were incubated in PBS at room temperature for 10 min to form the bOmCI-hC5 complex. Formation of the complex was confirmed by native PAGE gel shift (see Ref. 8 for example). Equal amounts bOmCI-hC5 or bOmCI alone were serially diluted in 75 μl of PBS before adding 75 μl of PBS containing ∼24,000 cpm ^3^H-LTB_4_. Following shaking incubation (3 h, 500 rpm, room temperature), samples were centrifuged at 8000 × *g* for 2 min, and the radioactivity remaining in solution was measured on a Wallac 1217 Rackbeta liquid scintillation counter after transferring 20 μl of the supernatant to 4 ml of Beckman Ready Value scintillation mixture. PBS only and serial dilutions of RaHBP2 and hC5 in PBS were negative controls.

##### Thrombin-mediated Cleavage of C5

One μg of hC5 and 2.5 NIH units of human thrombin were incubated in PBS at 37 °C for 1.5 h, alone, together, and with or without OmCI or protease inhibitor mixture (Sigma, catalog No. P8340). 1×, 10×, and 100× molar excess of OmCI to hC5 were used. Following incubation, human thrombin was inactivated (65 °C for 4 min), and free C5a was detected using a DRG Diagnostica enzyme immunoassay kit (catalog No. RE59292).

##### Immune Complex Acute Lung Injury Model

All animal experiments were approved by the CNRS institutional animal research committee and complied with the French Government's ethical and animal experiment regulations. C57/BL6 mice, male, about 8 weeks old and with a body weight of about 25 g, were dosed intranasally with 150 μg of chicken anti-ovalbumin IgG (anti-OVA) together with the drug of interest (bOmCI, bOmCI-LTB_4_, RaHBP2 or MK886) in 40 μl of saline. Immediately thereafter, immune complex in the lung was induced by injecting 300 μg of OVA and 0.3% Evans blue into the tail vein. Experiments were performed three times with groups of six animals in each treatment. MK886 was administered at 1 mg/kg by gavage and LTB_4_ by intranasal instillation. In the latter experiments, OmCI and RaHBP2 were administered immediately before LTB_4_. At 4 h after OVA or LTB_4_ administration, mice were killed, BAL was performed, and lungs were perfused with an isotonic solution and then excised. Cells in BAL were counted, and differential staining was performed using Diff-Quik. Vascular leakage was quantified by protein concentration in the bronchoalveolar space, and Evans blue was measured in BAL supernatant by absorbance at 460 nm. The excised lung was fixed in 4% buffered formaldehyde for H&E, and microscopic lesions (endothelial cell damage, inflammatory cell recruitment, and erythrocytes in the alveolar space) were assessed using a semiquantitative score (0–3) by two independent observers. C5a in the BAL supernatant was measured using the C5a micro MTPL EIA kit in accordance with the manufacturer's instructions (DRG Diagnostics GmbH).

##### Statistical Analyses

The data are presented as the mean ± S.D. or 95% CI as indicated in the figure legends. The significance of differences between two groups was determined by one-way analysis of variance (non-parametric test) using GraphPad Prism software, version 5, or in R using Tukey's multiple comparison of means. Plots of the residuals were used to confirm the homoscedasticity of the data. Statistical significance was reported if *p* was <0.05.

## RESULTS

### 

#### 

##### OmCI Binds a Variety of Unbranched Fatty Acids, but Its Preferred Ligand Is LTB_4_

**FIGURE 1.**
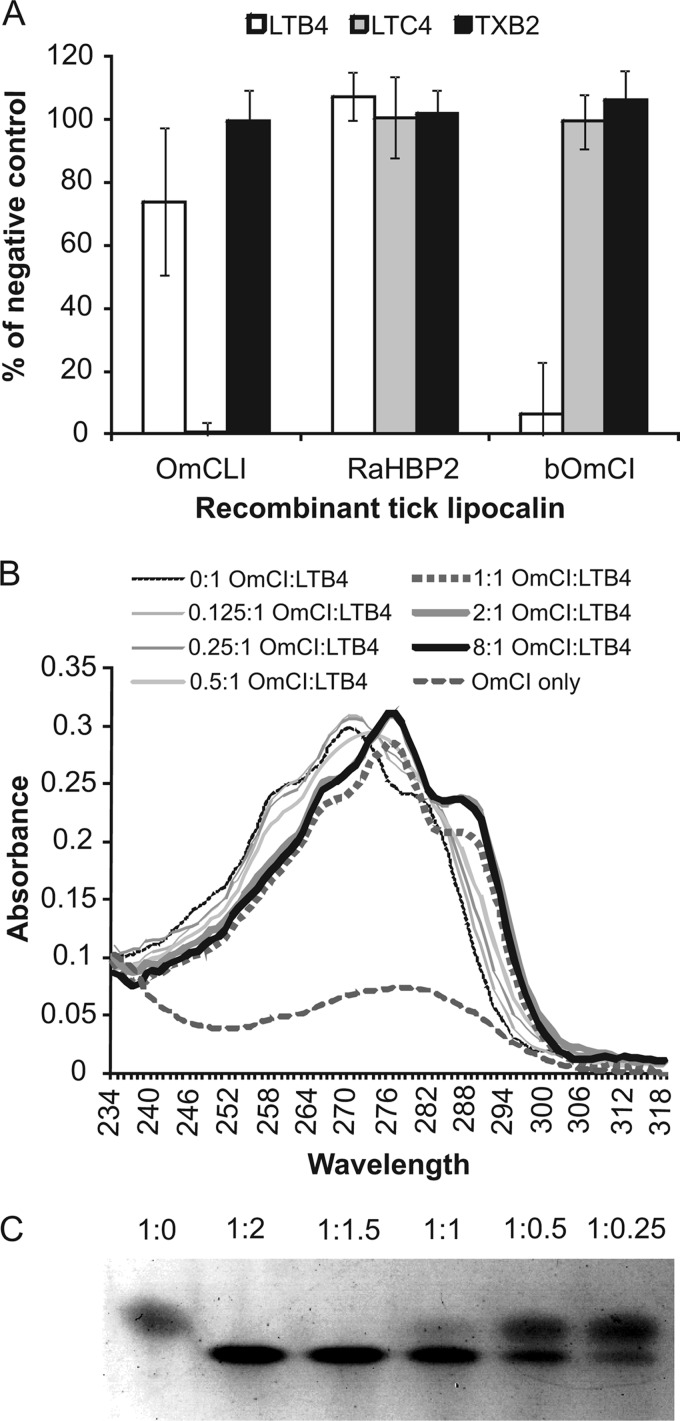
**OmCI binds LTB_4_.** *A*, bOmCI (5 μg) competes with LTB_4_-specific polyclonal Ab for binding to LTB_4_ but does not compete for LTC_4_ or TXB_2_. Negative control was PBS only. Tick lipocalin protein controls were 5 μg of OmCLI (mass, 16.9 kDa) and 5 μg of RaHBP2 (mass, 20.4 kDa). Shown are four replicates per sample from three independent experiments (95% CI). *B*, detection of LTB_4_ binding to OmCI shown by red shift in the characteristic absorbance spectra of the leukotriene. Peak absorbance shifts from 271 nm with shoulders at 261 and 281 nm for LTB_4_ only (*i.e.* molar ratio 0:1) to 277 nm with shoulders at 267 and 287 nm at LTB_4_ to bOmCI molar ratios of 1:1 and higher. For clarity, absorbance values measured at 1 nm intervals are shown as smoothed lines. *C*, LTB_4_ binding causes a mobility shift in bOmCI by Coomassie Blue-stained native PAGE. The molar ratio of bOmCI to LTB_4_ is shown above each *lane*.

We reported previously that OmCI expressed in yeast (yOmCI) is bound to ricinoleic acid (C_18_H_34_O_3_) and speculated that the natural host ligand bound by OmCI when secreted into the tick feeding site might be a proinflammatory eicosanoid (6). This was confirmed later using homologues of OmCI derived from other tick species (43). Solvent extraction and GC-MS analysis of the highly purified bOmCI used in this study (supplemental Fig. S1) showed that C_16:1_ *cis*-palmitoleic acid (C_16_H_30_O_2_) was the dominant (∼64%) fatty acid in the binding pocket of bOmCI, with various other C_16_–C_18_ linear fatty acids present in lower proportions (supplemental Fig. S2). Preferential binding to LTB_4_ was shown using an enzyme immunoassay in which bOmCI sequestered LTB_4_, preventing the AP-conjugated eicosanoid from interacting with specific capture antibodies of unknown, but presumably high, avidity (Fig. 1*A*). In the same assay, bOmCI did not show evidence of binding to the cyclic eicosanoid TXB_2_ (a stable derivative of TXA_2_) or to LTC_4_, which has amino acids conjugated to carbon 6 (C^6^) of the fatty acid chain (Fig. 1*A*). The positive and negative control tick-derived recombinant lipocalins bound LTC_4_ only (OmCLI- and cysLT-specific) or none (RaHBP2- and histamine-specific) of the fatty acids tested (Fig. 1*A*).

The distinctive absorption spectrum of LTB_4_ was exploited to show that when bound to OmCI, the UV maxima of LTB_4_ exhibits a +6 nm bathochromic (red) shift to 277, 267, and 287 nm (Fig. 1*B*). The shift was complete at a 1:1 molar ratio of OmCI to LTB_4_. Dispersion interactions between the conjugated leukotriene and nearby amino acids cause the shift, which is consistent with the triene chromophore of LTB_4_ being completely encompassed by OmCI. Spectral shifts in LTB_4_ were not seen when using OmCLI or RaHBP2 (supplemental Fig. S3*A*). The absorption intensity and bathochromic shift was not altered by a large molar excess of AA (60×) or 12(*S*)-HETE (40×), neither of which has triene chomophores, indicating that OmCI is highly specific for LTB_4_ (supplemental Fig. S3*B*).

LTB_4_ binding by bOmCI-palmitoleic acid, but not yeast-expressed yOmCI-ricinoleic acid, altered the proteins mobility by native PAGE (Fig. 1*C*); the shift was complete at a 1:1.5 molar ratio of OmCI to LTB_4_. This encouraged us to examine whether LTB_4_ induces conformational changes in OmCI that affect its C-inhibitory function.

##### Structural Analysis of bOmCI and bOmCI-LTB_4_

**FIGURE 2.**
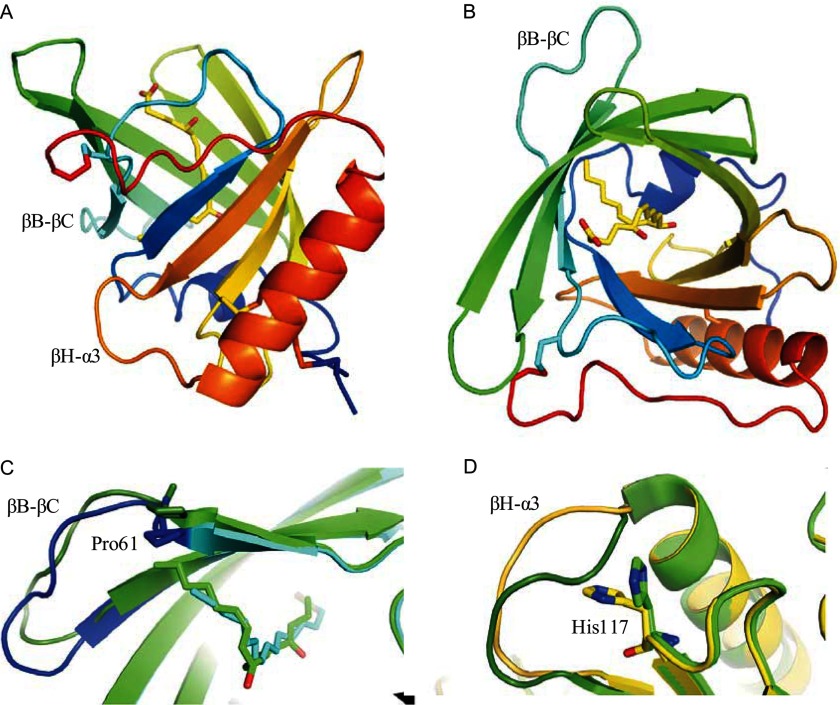
**Structure of bOmCI with and without LTB_4_ in the binding pocket.** *A* and *B*, schematic representations of the bOmCI molecule from the LTB_4_ cocrystal. *Blue* to *red*, from N to C terminus. The cysteine side chains and LTB_4_ ligand are shown in *stick* representation, with the ligand carbon atoms colored *yellow* and the oxygen atoms *red*. Views *A* and *B* are rotated forward by 90° around the horizontal axis. βB-βC and βH-α3 loops, which undergo conformational changes, are labeled. For the strand and helix labels see Ref. 6. *C* and *D*, representation of most significant, but still minor, conformational differences between bOmCI-palmitoleic acid and bOmCI-LTB_4_. *C*, the conformation of βB-βC loop-(61–70) changes with ligand size. *Green*, bOmCI-LTB_4_ complex; *blue*, bOmCI-palmitoleic acid complex; loop-(61–70), which swings out to better accommodate LTB_4_, is shown in *darker shades* in both molecules. The fatty acid ligands and Pro-61, the residue mediating the change in structure and contacting the ligand, are in *stick* representation. The yOmCI-ricinoleic acid complex is not shown, but its loop aligns with the conformation seen in the bOmCI-palmitoleic acid complex. *D*, His-117 and the βH-α3 loop (residues 135–141) are coupled. *Green*, bOmCI-LTB_4_ complex; *yellow*, yOmCI-ricinoleic acid (PDB accession code 1CM4). The βH-α3 loop (residues 135–141) is in *darker shades*. His-117, in which the side chain conformation accompanies the loop rearrangement, is represented in *sticks*.

We obtained two crystal structures of bOmCI, one in complex with palmitoleic acid and one in complex with LTB_4_ (PDB accession codes 3ZUI and 3ZUO). Both show the same overall structure as yOmCI-ricinoleic acid (PDB accession code 2CM4); root mean square deviation C_α_ = 0.61 and 0.63 Å for the palmitoleic acid and LTB_4_ complexes, respectively. Fig. 2, *A* and *B*, shows orthogonal views of bOmCI in complex with LTB_4_. The main ligand-dependent structural difference with respect to the yOmCI-ricinoleic acid crystal structure is the position of the loop βB-βC (residues 61–70), which pushes in/out to accommodate a smaller/larger ligand (see Fig. 2*C*). This movement is mediated by Pro-61, the residue at the bottom of the ligand pocket. The other main locus of structural variability in the bOmCI and yOmCI structures is the conformation of the βH-α3 loop (amino acids 132–142), shown previously to be required for C5 inhibition (43), which in both complexes of bOmCI swings inward, with the concomitant flip of the side chain of His-117 (Fig. 2*D*). However, the chemical nature of the ligand in the pocket bears no relationship to the conformation of the βH-α3 loop, as the loop was observed in both conformations in different copies of the yOmCI-ricinoleic acid complex (6).

**FIGURE 3.**
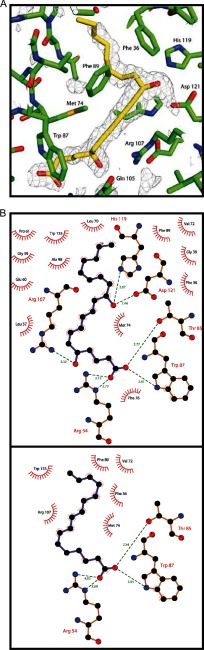
**Details of ligand binding.** *A*, bOmCI-LTB_4_ 1.9 Å *F_o_* − *F_c_* residual electron density contoured at the +1.0 σ level, is computed without modeling of the ligand. The final model for the LTB_4_ ligand is shown with carbon (*yellow*) and oxygen (*red*) surrounded by the OmCI pocket residues (carbon, *green*; oxygen, *red*; nitrogen, *blue*; and sulfur, *yellow*); this picture was produced with the program PyMOL (44). *B*, schematic representation of the bOmCI residues forming hydrogen bonds (with distances in Å) and non-bonding hydrophobic contacts to LTB_4_ (*upper panel*) and palmitoleic acid (*lower panel*). These pictures were produced with the program LigPlot (45).

The three ligands all bind with their carboxylic acid head at the solvent-exposed brim of the OmCI pocket, hydrogen bonding to the side chains of Arg-54, Thr-85, and Trp-87 (see Figs. 2, *A* and *B*, and 3). All ligands fit into the L-shaped pocket, with the kink at position C^12^ of LTB_4_, ricinoleic acid, and palmitoleic acid (Fig. 3). Hydroxylation at this position is common to ricinoleic acid and LTB_4_, and both complexes show the -OH group on C^12^ forming hydrogen bonds to His-119 and Asp-121 (Fig. 3*B, upper panel*). Palmitoleic acid lacks the -OH group at this position, and in that complex the same OmCI residues coordinate a water molecule instead. The ligands are also of different lengths (C_16_, C_18_, and C_20_, respectively, for palmitoleic acid, ricinoleic acid, and LTB_4_); LTB_4_ fills the pocket completely, whereas the yOmCI-ricinoleic acid and bOmCI-palmitoleic acid complexes show a water molecule filling the bottom of the pocket. The hydrophobic side chains of Phe-36, Tyr-43, Leu-57, Gly-59, Pro-61, Leu-70, Val-72, Met-74, Phe-76, Trp-87, Phe-89, Arg-107, and Trp-133 contact the hydrophobic body of all three ligands (Fig. 3*B*). The C_9_=C_10_ double bond present in LTB_4_, but not in palmitoleic or ricinoleic acid, stacks against the guanidinium head of Arg-107. The same Arg side chain is the source of the selectivity against cyclic eicosanoids such as TXB_2_ and TXA_2_ (43), as it makes the pocket too narrow to accommodate cyclic ligands. Gln-105 recognizes the -OH at LTB_4_ C5. The OmCI pocket is predicted to be unable to accommodate -OH at C15 because of the side chains of Phe-36 and Tyr-43, so the anti-inflammatory eicosanoids 15(*S*)HETE, lipoxin A_4_, and lipoxin B_4_ are unlikely to be bound by OmCI.

##### The LTB_4_ and C5 Binding Activities of OmCI Are Independent

**FIGURE 4.**
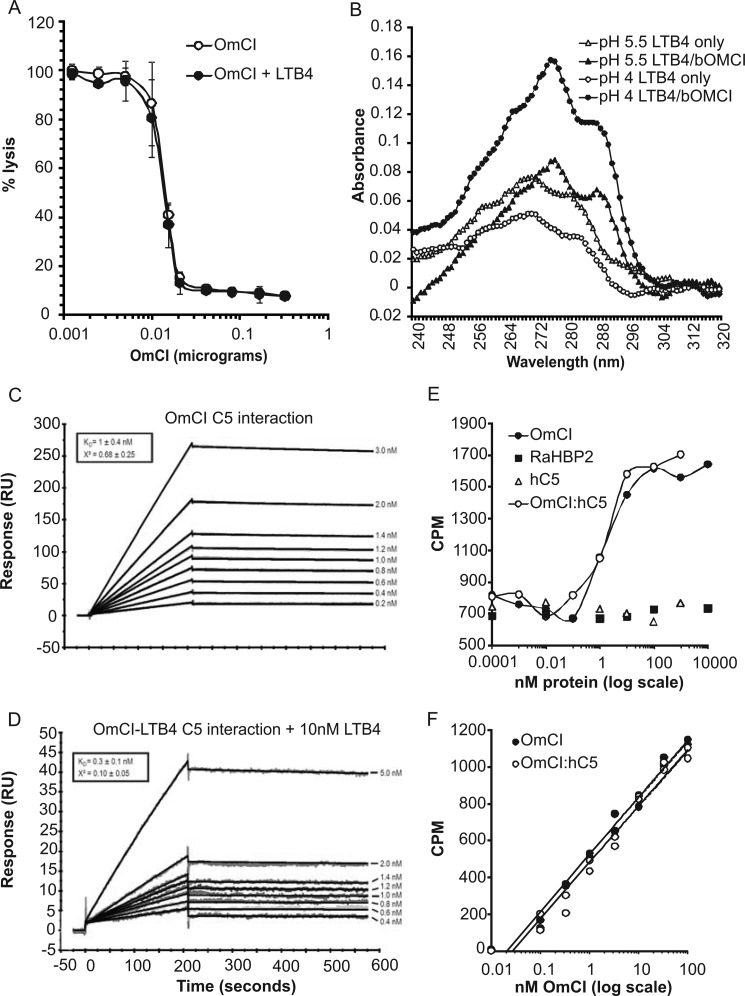
**C5 and LTB_4_ binding activities of OmCI are independent.** *A*, LTB_4_ (2-fold molar excess) has no effect on the classical C pathway inhibitory activity of bOmCI assayed by hemolysis. *Bars* show 95% CI (*n* = 4 for each data point). *B*, spectroscopy showing that LTB_4_ remains bound (characteristic red-shifted spectrum) to bOmCI in low pH (5.5 and 4), 10 mm sodium acetate buffers. *C* and *D*, SPR measurement of binding between bOmCI and human C5 (hC5) in the absence (*C*) and presence (*D*) of LTB_4_. Curves were fitted using a 1:1 Langmuir model (shown in *black*). Mean *K_D_* and chi-squared values are shown with their standard deviation calculated from 15 replicates. Replicates were performed over a minimum of two independent experiments. The RUs in *D* are lower than in *C* because less bOmCI-LTB_4_ than bOmCI coupled to the sensor surface. *E* and *F*, binding of radiolabeled LTB_4_ by bOmCI alone and in complex with hC5. *E*, plot showing that ligand binding is saturable. RaHBP2 and hC5 alone do not show saturable binding, and cpm values are equivalent to PBS only (not shown). Raw data shown (*n* = 2 for each data point) are representative of three independent experiments. The highest concentration of OmCI-hC5 (*i.e.* 10000 nm) was not assayed, as the amount of hC5 required was prohibitively expensive. *F*, plot showing interaction of hC5 with bOmCI has no effect on the binding kinetics to LTB_4_. The data are representative of two independent experiments (*n* = 2 for each data point) and show cpm values after subtraction of average cpm (*n* = 16) of the negative control (RaHBP2). Logarithmic regression line functions are: bOmCI, *y* = 134.67 ln(*x*) + 521.6, *R*^2^ = 0.98; bOmCI-hC5, *y* = 132.87 ln(*x*) + 479.2, *R*^2^ = 0.97.

The subtle conformational change induced by LTB_4_ binding may have an effect on the binding of OmCI to C5. Conversely, C5 binding may change the binding affinity of OmCI for LTB_4_. We tested both possibilities by examining the binding affinity between purified hC5 and bOmCI, with or without LTB_4_, and the binding kinetics of OmCI for ^3^H-labeled LTB_4_ in the presence of excess hC5. We first showed that excess LTB_4_ had no effect on the potency of bOmCI measured by classical hemolytic assay (Fig. 4*A*). Before undertaking SPR experiments, we confirmed that LTB_4_ remains bound to bOmCI at the low pH 4.5 needed to couple the protein to the chip (Fig. 4*B*), and where applicable, LTB_4_ was also included in the running buffer. The SPR data showed no physiologically relevant change in hC5 affinity with and without LTB_4_ (Fig. 4, *C* and *D*: *K_D_* 1 nm ± 0.4 without LTB_4_ and 0.3 nm ± 0.1 nm with LTB_4_). We considered the difference physiologically irrelevant because the concentration of C5 in human serum is ∼0.4 μm, and because both *K_D_* values were more than 40 times tighter than this concentration, OmCI would be present as a complex with C5 whether or not LTB_4_ was bound. The *K_D_* calculated here by SPR for the interaction between bOmCI and hC5 is lower (tighter) than that reported previously (7) for yOmCI (*K_D_* 18.5 nm) but is characterized by a similar very slow off-rate. yOmCI has two site-directed point mutations that prevent hyperglycosylation when expressed in yeast, which may alter the binding kinetics. Radioligand assays show no alteration of LTB_4_ binding in the presence or absence of hC5 (Fig. 4, *E* and *F*). OmCI and preformed OmCI-hC5 complex show equivalent saturable binding to ^3^H-LTB_4_, whereas PBS (not shown but equivalent to the following two protein controls), RaHBP2 (specific for histamine), and hC5 do not (Fig. 4*E*). The assay measured the ability of OmCI to bind ^3^H-LTB_4_ and keep it in solution. Indeed, no more than 20% of the labeled LTB_4_ remained in solution in the negative control samples, whereas more than 50% remained in solution at the higher concentrations of OmCI (Fig. 4*E*), confirming that OmCI binding is needed for solubilization. Because of the low solubility of LTB_4_ in this assay, association and dissociation constants could not be accurately derived. However, a comparison of the slope of the logarithmic regression functions for equivalent concentrations of OmCI and OmCI-hC5 indicate that the binding kinetics between LTB_4_ and OmCI are not altered when OmCI is in complex with hC5 (Fig. 4*F*). In summary, despite a subtle structural change (Fig. 2*C*) caused by binding to OmCI, LTB_4_ has no physiologically relevant effect on binding between OmCI and C5, and conversely, C5 binding by OmCI does not hinder or enhance binding to LTB_4_. This result is in agreement with previous suggestions that C5 binding and entry of LTB_4_ to the binding pocket happen on opposite faces of OmCI (6, 46).

##### OmCI Ameliorates IC-ALI in Mice

**FIGURE 5.**
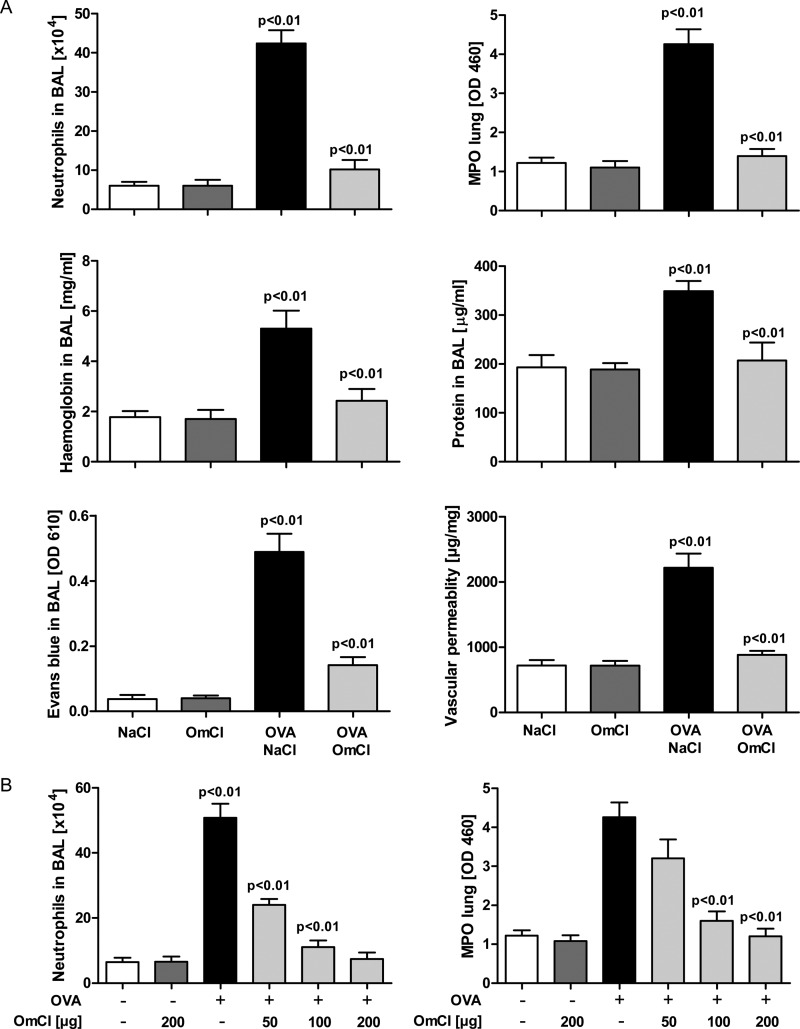
**OmCI inhibits IC-ALI.** *A*, OmCI (100 μg) reduces neutrophils in BALF and lung (myeloperoxidase activity) and prevents hemorrhage, protein and Evan's Blue (*EB*) leak, and edema. *B*, dose-response effect of OmCI (50, 100, and 200 μg/mouse) on neutrophil recruitment. All treatment groups except saline (NaCl) were dosed intranasally with chicken anti-OVA IgG with or without OmCI. All groups then received OVA by injection into the tail vein. Lung inflammation was evaluated 4 h after induction of IC. The mean values + S.D. of a representative study are given (*n* = 6 mice/group).

**FIGURE 6.**
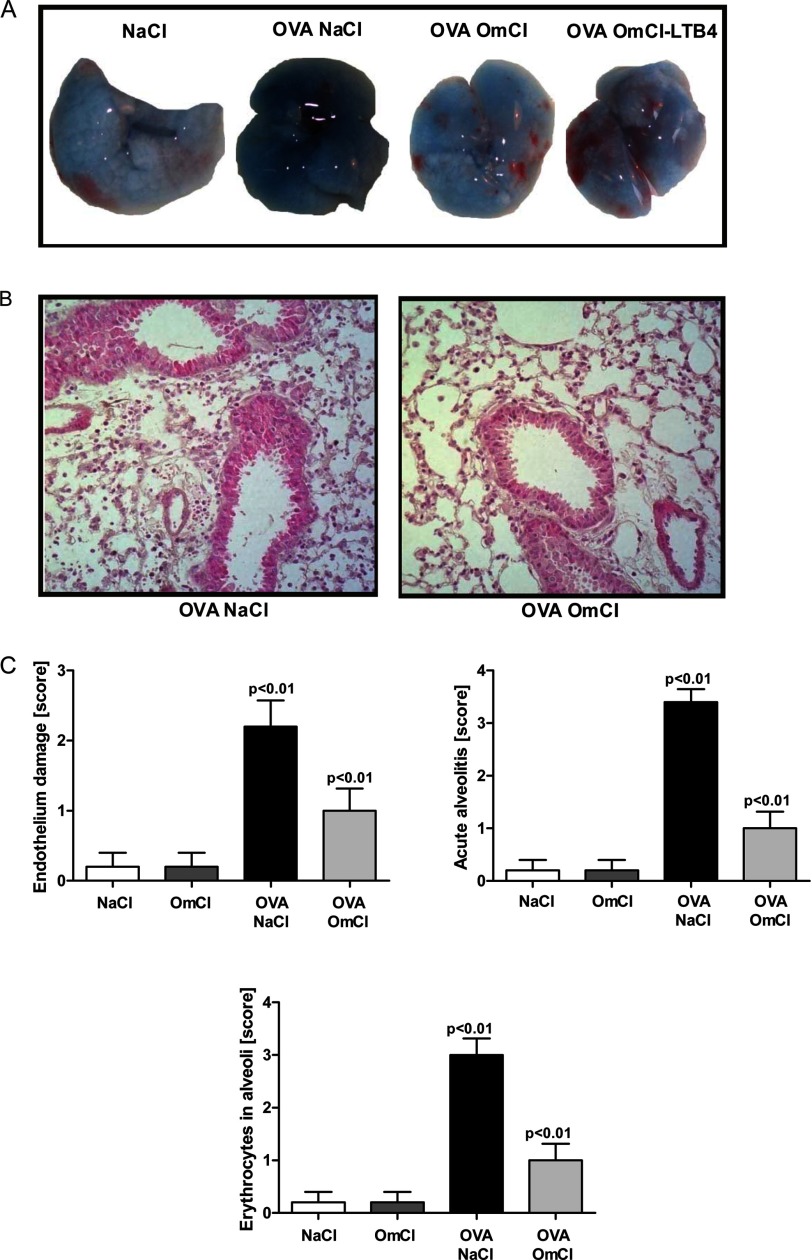
**OmCI (100 μg) inhibits vascular leak, tissue damage, and inflammation in response to IC.** *A*, OmCI attenuates vascular leak as shown by *blue* macroscopic discoloration due to Evans blue leakage. OmCI+LTB_4_ signifies OmCI saturated with LTB_4_ before its addition to lung. *B*, OmCI inhibits endothelial damage, neutrophil recruitment, and hemorrhage into the alveolar space (H&E staining, magnification ×100). *C*, semiquantitative score of endothelial damage, neutrophil recruitment, and hemorrhage in the lung. Acute inflammation was evaluated at 4 h. Representative data for one of three independent experiments are shown (*n* = 6 mice/group). Values are shown as mean + S.D.

Based on our understanding of the binding activities of OmCI, we selected the well characterized mouse model of IC-ALI (33) to test the therapeutic potential of the protein. In this pathology, activation of C and IgG FcγR on effector cells, chiefly alveolar macrophages, are the dominant initial events following immune complex formation (9, 33). C5a, acting through C5aR signaling, up-regulates the expression of activating FcγRIII and down-regulates inhibitory FcγRIIB IgG receptors (35, 36). The FcγR imbalance leads to IC-ALI via alveolar macrophage production of proteases, early response cytokines (TNFα and IL-1β), and LTs (predominantly LTB_4_ (47)), which with C5a increase the expression of adhesion molecules on vascular endothelial cells, and induce CXCR1/2 chemokines. These in turn recruit abundant neutrophils that degranulate and release superoxides, causing inflammation and pulmonary microvascular damage (9). We first showed that OmCI itself does not induce an inflammatory response when administered intranasally (Fig. 5*A*, compare OmCI and saline). As hoped, bOmCI profoundly inhibited immune complex-induced neutrophil recruitment to lung tissue (measured by myeloperoxidase) and BALF (Fig. 5*A*). It also decreased pulmonary microvascular damage, with significantly reduced hemorrhage, edema, and protein exudation to the bronchoalveolar space (Fig. 5*A*). At 100 μg/mouse, bOmCI reached a plateau for near complete inhibition of myeloperoxidase activity and neutrophil recruitment to the alveolar space, with no further decrease at the 200-μg dose (Fig. 5*B*). Macroscopically a distinct capillary leak of Evans blue from the blood to the lung can be seen, which is attenuated by OmCI (Fig. 6*A*). Representative microscopic plates and semiquantitative microscopic evaluation show that bOmCI reduced endothelial cell damage, inflammation, and erythrocyte extravasation into the bronchoalveolar space (Fig. 6, *B* and *C*).

##### Inhibition of Both C5 Activation by C and LTB_4_ Binding Is Needed for Full Inhibitory Effect of OmCI

**FIGURE 7.**
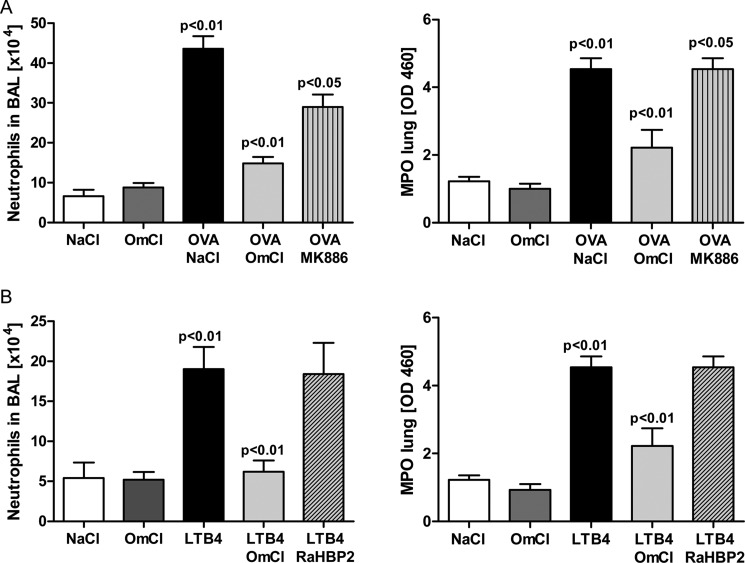
**OmCI (100 μg) neutralizes LTB**_4_**-induced inflammation.** *A*, inhibition of leukotriene biosynthesis by MK886 (1 mg/kg by gavage) reduces neutrophil recruitment induced by IC-ALI. *B*, intranasal instillation of LTB_4_ (1 μg) induces neutrophil recruitment in the lung, which is inhibited by prior administration of 2-fold molar excess bOmCI but not by a similar amount of control tick lipocalin histamine-binding protein RaHBP2 (100 μg). Representative data for one of three independent experiments are shown (*n* = 6 mice/group). Values are shown as mean + S.D.

The proven independence of the two binding activities of OmCI enabled us to investigate the relative importance of LTB_4_ and C5 activation in IC-ALI. IC-ALI has been investigated intensively by many research groups, but the role of LTB_4_ in IC-ALI has been overlooked by all but one group (48–50). We therefore first confirmed that LTB_4_ had a functional role in our model. In agreement with the earlier work (48, 49), we found that 4 h after administration of OVA, LTB_4_ levels in BAL increase significantly (*p* < 0.01) in response to immune complex: saline control 125.8 pg/ml LTB_4_ (95% CI, 94.7–156.9 pg/ml, *n* = 8), OVA 243.6 pg/ml LTB_4_ (95% CI, 292.8–194.45 pg/ml, *n* = 8). Furthermore, administration of MK886, which prevents biosynthesis of LTs, significantly reduced vascular leakage (data not shown) and inflammation, although the inhibitory effect of MK886 was less pronounced than that because of bOmCI (Fig. 7*A*). Intranasal administration of LTB_4_ (1 μg) caused a significant increase in neutrophil recruitment to both the lung tissue and the bronchoalveolar space (Fig. 7*B*). Neutrophil recruitment in response to exogenous LTB_4_ was inhibited significantly by prior administration of bOmCI (in 2-fold molar excess to LTB_4_) and was not inhibited by prior administration of an equal amount of histamine-specific RaHBP2 (Fig. 7*B*), suggesting that OmCI binds LTB_4_ within the lung.

**FIGURE 8.**
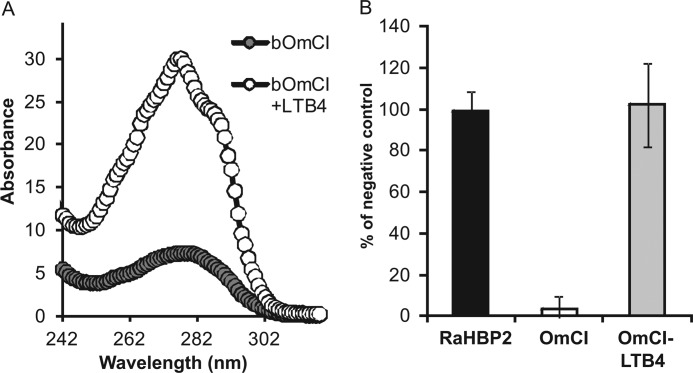
**OmCI saturated with LTB**_4_ **(bOmCI-LTB**_4_**) is unable to bind additional LTB_4_.** *A*, representative absorption spectrum of bOmCI with and without LTB_4_ that was used for experiments in mice, crystallization, and SPR. *B*, unlike bOmCI, in an enzyme immunoassay bOmCI-LTB_4_ is unable to compete with LTB_4_-specific polyclonal Ab for binding to LTB_4_. Negative control was PBS only. A 6-fold excess (30 μg; see legend for Fig. 1*A*) of each protein, including the histamine-binding protein 2 (RaHBP2), was used for each replicate. Four replicates/sample (95% CI) are shown.

**FIGURE 9.**
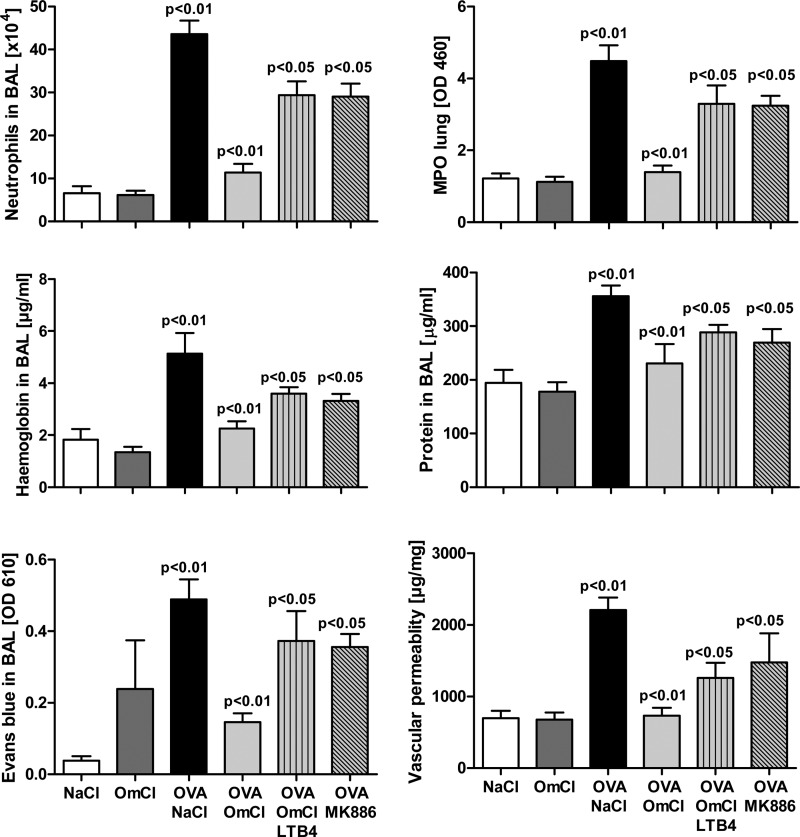
**Saturation of OmCI with LTB_4_ (OmCI-LTB**_4_**) reduces the inhibitory effect of the lipocalin on IC-ALI.** Representative data for one of three independent experiments are shown (*n* = 6 mice/group). Values are shown as mean + S.D.

**FIGURE 10.**
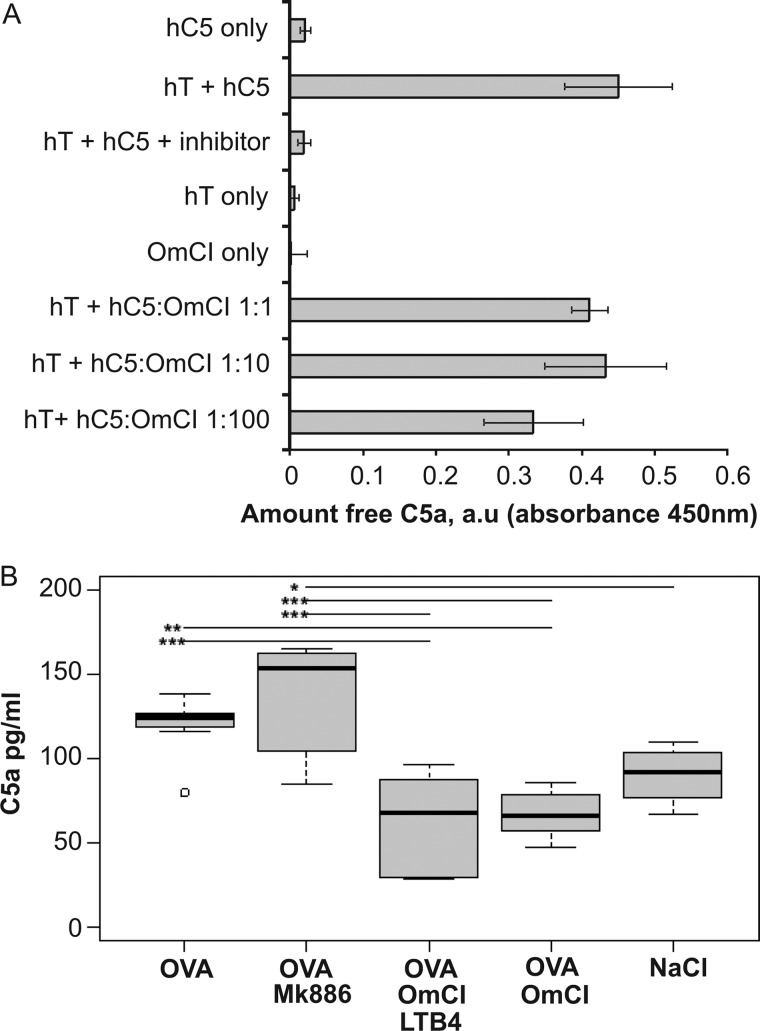
**OmCI does not inhibit cleavage of C5a from thrombin but does reduce the concentration of C5a in the alveolar space.** *A*, assay of the inhibitory effect of OmCI on cleavage of C5a from hC5 by human thrombin (*hT*). The average background value (*i.e.* PBS only, blank wells) was subtracted from all samples. Four replicates/sample (95% CI) are shown. Combined data from two independent experiments are shown. *B*, C5a concentration in BALF in each treatment group (*n* = 8 mice/group) measured at 4 h after induction of IC-ALI. The box plot shows median values, upper and lower quartiles, largest and smallest observations for each group, and the single outlier in the OVA group. Significant differences between groups, determined by analysis of variance using Tukey's multiple comparison of means, are indicated by *asterisked bars* (*, *p* < 0.02; **, *p* < 0.005; ***, *p* < 0.001).

To examine the importance of each proinflammatory mediator, bOmCI was saturated with LTB_4_ to form bOmCI-LTB_4_ (Fig. 8*A*), and the *in vivo* activity was compared with parent bOmCI. We first showed that LTB_4_-saturated bOmCI cannot bind additional LTB_4_ (Fig. 8*B*) but binds and inhibits C5 as potently as bOmCI (Fig. 4, *A*, *C*, and *D*). bOmCI-LTB_4_ significantly attenuated all the parameters measured but the anti-inflammatory effect was less marked than that of bOmCI with binding capacity for LTB_4_ (Figs. 9 and Fig. 6*A*, *rightmost* lung). In agreement with its known binding locality remote from the C5a cleavage site (6, 8), OmCI does not prevent thrombin mediated cleavage of C5a from C5 (Fig. 10*A*), therefore the inhibitory activity of bOmCI-LTB_4_ seen in IC-ALI is likely due to inhibition of C5 activation solely by the C5 convertase and not by non-C proteases released by activated alveolar macrophages (18, 19). Both OmCI and OmCI saturated with LTB_4_ significantly decreased the concentration of C5a in BALF measured at 4h compared with mice treated with OVA alone, or OVA and MK886 which prevents LTB_4_ synthesis but has no direct action on C5 (Fig. 10*B*).

## DISCUSSION

The tick derived bifunctional protein OmCI significantly attenuates the symptoms of experimental IC-ALI. Furthermore, inhibition of C-mediated C5 activation and binding of LTB_4_ are both required for full potency of OmCI in this animal model. Our biochemical and structural data show that this is not mediated by an allosteric effect on OmCI, since C5 has no effect on LTB_4_ binding, and although LTB_4_ increases the binding affinity of OmCI for C5 measured by SPR this is unlikely to have any physiological relevance on the inhibitory activity of the protein *in vivo*.

The importance of C5a in driving IC-ALI through modulation of FcγR signaling in response to immune complex is well established (33, 35, 36). Current literature on this pathology emphasizes a dominant role for C5 activation within the alveolar space via non-C5 convertase serine proteases (including thrombin) released from alveolar macrophages in response to immune complex (9, 18, 19, 33). However, the significant inhibitory activity of bOmCI saturated with LTB_4_ (bOmCI-LTB_4_), which specifically inhibits C5 activation by classical and alternative C5 convertases (C3bC2C3b and C3bBbC3b, respectively) and has no effect on C5 activation by non-C proteases, indicates that C5 activation directly mediated by C5 convertase, which is present and active in BALF (51, 52), has a significant functional role in IC-ALI. This conclusion is supported by earlier work, which showed that thrombin inhibitors significantly ameliorate IC-ALI in C3^−/−^ mice with supernormal levels of thrombin but not C3^+/+^ mice with functional C and normal levels of thrombin (18). Thus non-C protease-mediated cleavage of C5 probably has a secondary role in amplification of C5a formation in C-sufficient mice.

With the focus on C5a, the importance of LTB_4_ in IC-ALI has largely been overlooked by all but one research group (48–50), although the pivotal role of LTB_4_ in other models of lung disease such as LPS-induced ALI- and allergen-induced airway hypersensitivity is well established (13, 53, 54). Our work supports the earlier work showing that LTB_4_ plays a significant part in the pathology of IC-ALI (49, 50). Furthermore, we find that LTB_4_ inhibition is as important as C convertase C5 activation for amelioration of symptoms of IC-ALI in the mouse model. This highlights the co-dependent effects of C5 and LTB_4_ on polymorphonuclear leukocyte migration and recruitment and other proinflammatory processes (9, 13–15, 21–24). It seems likely that previous work reporting a dominant role for C5 (32–37) may well have reduced LTB_4_ synthesis, thereby preventing its action on the lung; however, the role of LTB_4_ was not examined in these studies.

We find it interesting that natural selection has not excluded binding of a variety of unbranched fatty acids by ectoparasite-derived OmCI. To date these are known to include palmitoleic acid and related C_16_-C_18_ fatty acids and ricinoleic acid (C_18_) and the preferred ligand LTB_4_ (C_20_). The closely related tick lipocalin, TSGP3, shows even greater promiscuity, binding cyclic eicosanoids (here carbocyclic TXA_2_) as well as LTs (43). Both TSGP homologues and presumably OmCI also show moderately high affinity binding (*K_D_* ∼60 nm) to AA (43). We speculate that by sequestering AA and possibly LTA_4_ (the immediate precursor of LTB_4_), at sites of inflammation such as the tick feeding site, OmCI may decrease transcellular synthesis from AA (14, 55) of LTB_4_ and other proinflammatory (cysLTs and TXA_2_) or LT-enhancing eicosanoids such as prostaglandins. Thus, in addition to inhibiting TCC and signaling via the BLT1, BLT2, C5aR, and C5L2 receptors, OmCI may decrease the overall rate of eicosanoid synthesis at inflammatory sites.

**FIGURE 11.**
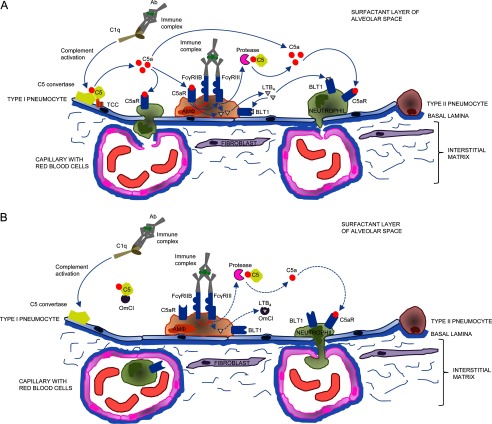
**Interactions and feedback mechanisms between OmCI, complement, Fcγ receptors, and LTB**_4_ **in early phase of mouse IC-ALI.** *A*, simplified view of co-dependent proinflammatory pathways focused on alveolar macrophages (*AM*Φ) in the alveolar space in the absence of OmCI. *B*, the same pathways as in *A* with OmCI present. For clarity, inflammatory mediators stimulated by TCC, C5a, and LTB_4_ and cellular responses within the lung are not shown. Note that only the LTB_4_ receptor BLT1 is shown, as BLT2 expression in mice is restricted to mast cells and keratinocytes. The C5a receptor C5L2 is omitted from the figure, as its actions are subject to debate. *Blue arrow*, stimulatory pathway; *red line*, inhibitory pathway. *Solid line*, full activation of pathway; *dotted line*, partial activation of pathway.

Our current hypothesis to explain the potent activity of OmCI in IC-ALI is that intranasally administered OmCI prevents cleavage of C5 by C5 convertases on alveolar epithelial surfaces activated via IC, thereby limiting TCC formation and C5a activation of alveolar macrophages and other cells via FcγR threshold modulation. We propose that C convertase-mediated cleavage of C5 initiates the activation of alveolar macrophages, which subsequently increases the release of proteases that activate C5 independently of C. Separately, sequestration of LTB_4_ by OmCI limits the action of both convertase and non-C protease (*e.g.* thrombin)-derived C5a, which recruit leukocytes, and in particular tissue damaging neutrophils, both directly through C5aR and via the LTB_4_ receptors BLT1 and BLT2 (22, 56). The direct and indirect actions of these co-dependent systems (15, 21–24, 57) are summarized in Fig. 11. At present we do not know whether intranasally administered OmCI is able to enter tissue and prevent the action of C and LTB_4_ within the lung or whether it acts entirely at the epithelial surface. Future studies should examine the effect of OmCI on FcγR and additional pro- and anti-inflammatory mediators and clarify the site at which OmCI acts, as this may have a direct bearing on the optimal route of administration. Investigating the elaboration of the immune response via chemokines and cytokines induced by C5 activation and LTB_4_ using OmCI mutants able to bind only C5 or LTB_4_ might more fully elucidate the downstream effects of these two mediators.

In conclusion, our data highlight the need to evaluate the functions of LTB_4_, and possibly other eicosanoids, more closely in IC-ALI and to reevaluate the relative importance of C5a derived by complement-dependent and -independent pathways in C3-sufficient mice. The data show that dual inhibition of C5 activation and LTB_4_ can have a profound inhibitory effect on inflammation and in particular on neutrophil recruitment and activation. Therapeutic indications for OmCI, or other combined inhibitors of C5 and LTB_4_, may include acute forms of immune complex-mediated diseases with neutrophil-dependent tissue injury, such as transfusion-related ALI, sepsis, rheumatic fever, and some forms of rheumatoid arthritis, peritonitis, and glomerulonephritis (58). The potential therapeutic value of dual acting drugs is widely recognized by the pharmaceutical industry, where there is currently intense interest in bispecific Ab (59). Notably, the functional independence of the two binding activities of OmCI and the detailed structural understanding of fatty acid binding that we have described will enable protein engineering for the selective targeting of eicosanoids most relevant to treatment of specific pathologies.

## Supplementary Material

Supplemental Data

## Footnotes

8 The abbreviations used are:

LTB_4_: leukotriene B4
LT: leukotriene
C: complement
TCC: terminal 5b-9 C complex
AA: arachidonic acid
cysLT: cysteinyl leukotriene
IC-ALI: immune complex-induced acute lung injury
hC5: human C5
OmCI: *Ornithodoros moubata* complement inhibitor
bOmCI: bacterially expressed OmCI
yOmCI: OmCI expressed in yeast
OmCLI: *Ornithodoros moubata* cysteinyl leukotriene inhibitor
RaHBP2: *Rhipicephalus appendiculatus* histamine-binding protein 2
12(*S*)-HETE: 12(*S*)-hydroxyeicosatetraenoic acid
TXA_2_: thromboxane A_2_
TXB_2_: thromboxane B_2_
AP: alkaline phosphatase
PDB: Protein Data Bank
OVA: ovalbumin
BAL: bronchoalveolar lavage
BALF: bronchoalveolar lavage fluid
SPR: surface plasmon resonance.
