# Supplementary material for: Bifunctional Lipocalin Ameliorates Murine Immune Complex-induced Acute Lung Injury
Source: J Biol Chem. 2013 Apr 26;288(26):18789–802. doi: 10.1074/jbc.M112.420331 (PMC3696655; doi:10.1074/jbc.M112.420331)
Supplement: Supplemental Data [file supp_288_26_18789__index.html]

Bifunctional Lipocalin Ameliorates Murine Immune Complex-induced Acute Lung Injury — Dual Acting Inhibitor Ameliorates Lung Disease — Supplemental Data 

# Bifunctional Lipocalin Ameliorates Murine Immune Complex-induced Acute Lung Injury

## Supplemental Data

**Files in this Data Supplement:**

- Supplemental Figures 1, 2 and 3 (.pdf, 199 KB) - Fig 1. Purity of OmCI by RP-HPLC and PAGE. Fig. 2 GC-MS analysis showing that palmitoleic acid is the dominant fatty acid ligand in the binding pocket of bOmCI. Fig. 3 Spectroscopy of LTB4 in presence of tick lipocalins and eicosanoids.
- Supplemental Tables 1 and 2 (.pdf, 33 KB) - Table 1 bOmCI data collection and processing Table 2 bOmCI structure refinements statistics
