# Supplementary material for: Bifunctional Lipocalin Ameliorates Murine Immune Complex-induced Acute Lung Injury
Source: J Biol Chem. 2013 Apr 26;288(26):18789–802. doi: 10.1074/jbc.M112.420331 (PMC3696655; doi:10.1074/jbc.M112.420331)
Supplement: Supplemental Data [file supp_M112.420331_JBC_supplementary_Figures.pdf]

SUPPLEMENTARY FIGURE LEGENDS

FIGURE S1

Purity of OmCI by RP-HPLC and PAGE. A. Purified bOmCI was analyzed on a Shimadzu HPLC LC20 with a YMC-Pack C8, 200 Å, 3 µm, 250 x 4.6mm OC20S03-2546WT. Gradient elution was performed from 24 to 28 % acetonitrile with 0.0425% TFA at a flow rate of 1.0 ml/min at 50 °C. Injection volume was 1µl. Detection was performed by UV (214 nm). Comparison with reference OmCI and mass spectometry (not shown) identified the peak as OmCI. B. Representative Coomassie Blue stained 4-12% denaturing PAGE (Novex) showing purification of OmCI from bacterial supernatant.

FIGURE S2

GC-MS analysis showing that C-16 palmitoleic acid ($C_{16}H_{30}O_2$) is the dominant fatty acid ligand present in the binding pocket of bOmCI. A. Gas chromatography and mass spectrometry after extraction from a bOmCI sample and esterification identified the methyl ester of palmitoleic acid as the major product (64%); minor components were identified as elaidic acid methyl ester (25.6%), C17:1 *trans* methyl ester (7.6%) and oleic acid methyl ester (2.7%). B. The presence of palmitic- and elaidic acid methyl esters was confirmed by comparison with reference samples.

FIGURE S3

Spectroscopy of LTB_4 in presence of tick lipocalins and eicosanoids. A LTB_4 shows no signs of binding (no red shift in spectrum) to tick lipocalins RaHBP2 and OmCLI. B. Excess AA (60X) and 12(S)-HETE (40X) have no effect on the red shift caused by LTB_4 binding to bOmCI.

Figure S1

A

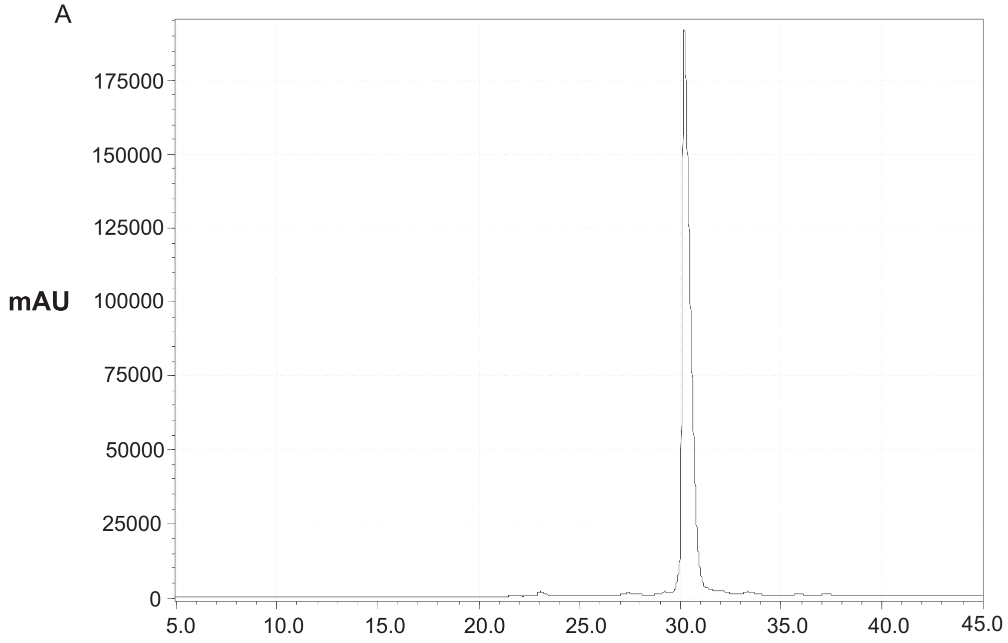

B

Lane	Sample
1	Mark12 (invitrogen)
2	Interim Standard
3	[1-1] Supernatant
4	[1-3] Filtrate
5	[2-3] QBB eluate
6	[3-4final] DEAE pool
7	[4-4final] HIC pool
8	[6-1] Filled API
9	Mark12 (invitrogen)

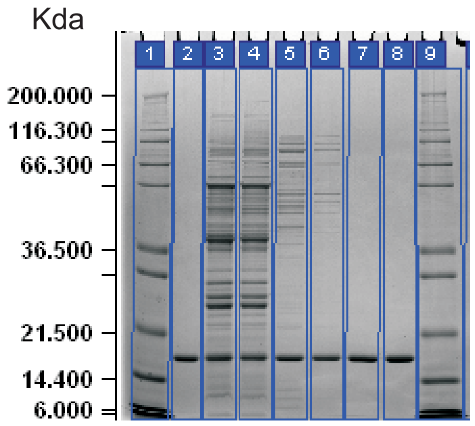

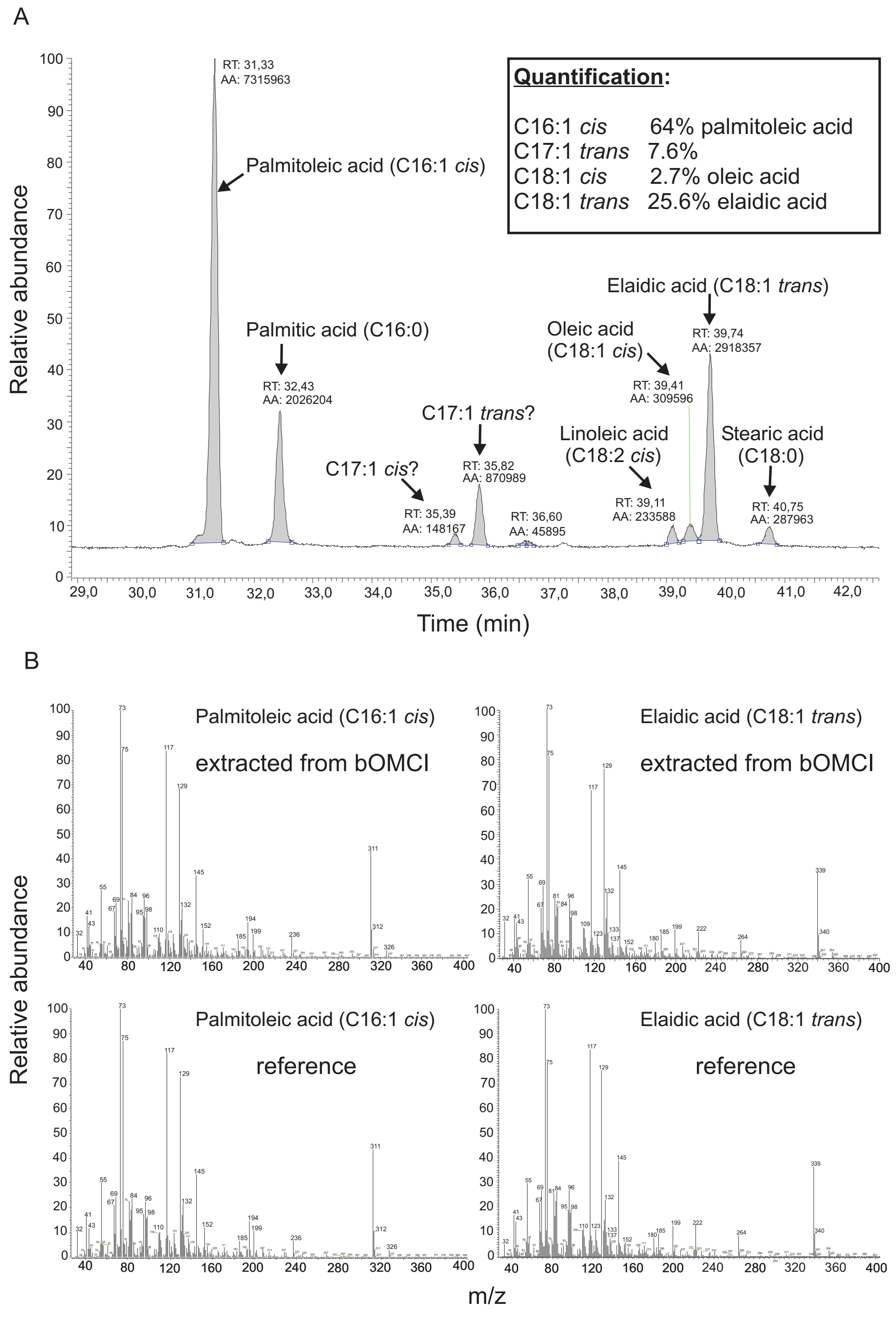
A
Quantification:
C16:1 cis 64% palmitoleic acid
C17:1 trans 7.6%
C18:1 cis 2.7% oleic acid
C18:1 trans 25.6% elaidic acid
RT: 31,33
AA: 7315963
Palmitoleic acid (C16:1 cis)
Palmitic acid (C16:0)
RT: 32,43
AA: 2026204
C17:1 cis?
RT: 35,39
AA: 148167
C17:1 trans?
RT: 35,82
AA: 870989
RT: 36,60
AA: 45895
Linoleic acid
(C18:2 cis)
RT: 39,11
AA: 233588
Oleic acid
(C18:1 cis)
RT: 39,41
AA: 309596
Elaidic acid (C18:1 trans)
RT: 39,74
AA: 2918357
Stearic acid
(C18:0)
RT: 40,75
AA: 287963
Relative abundance
Time (min)
B
Palmitoleic acid (C16:1 cis)
extracted from bOMCI
Elaidic acid (C18:1 trans)
extracted from bOMCI
Palmitoleic acid (C16:1 cis)
reference
Elaidic acid (C18:1 trans)
reference
Relative abundance
m/z

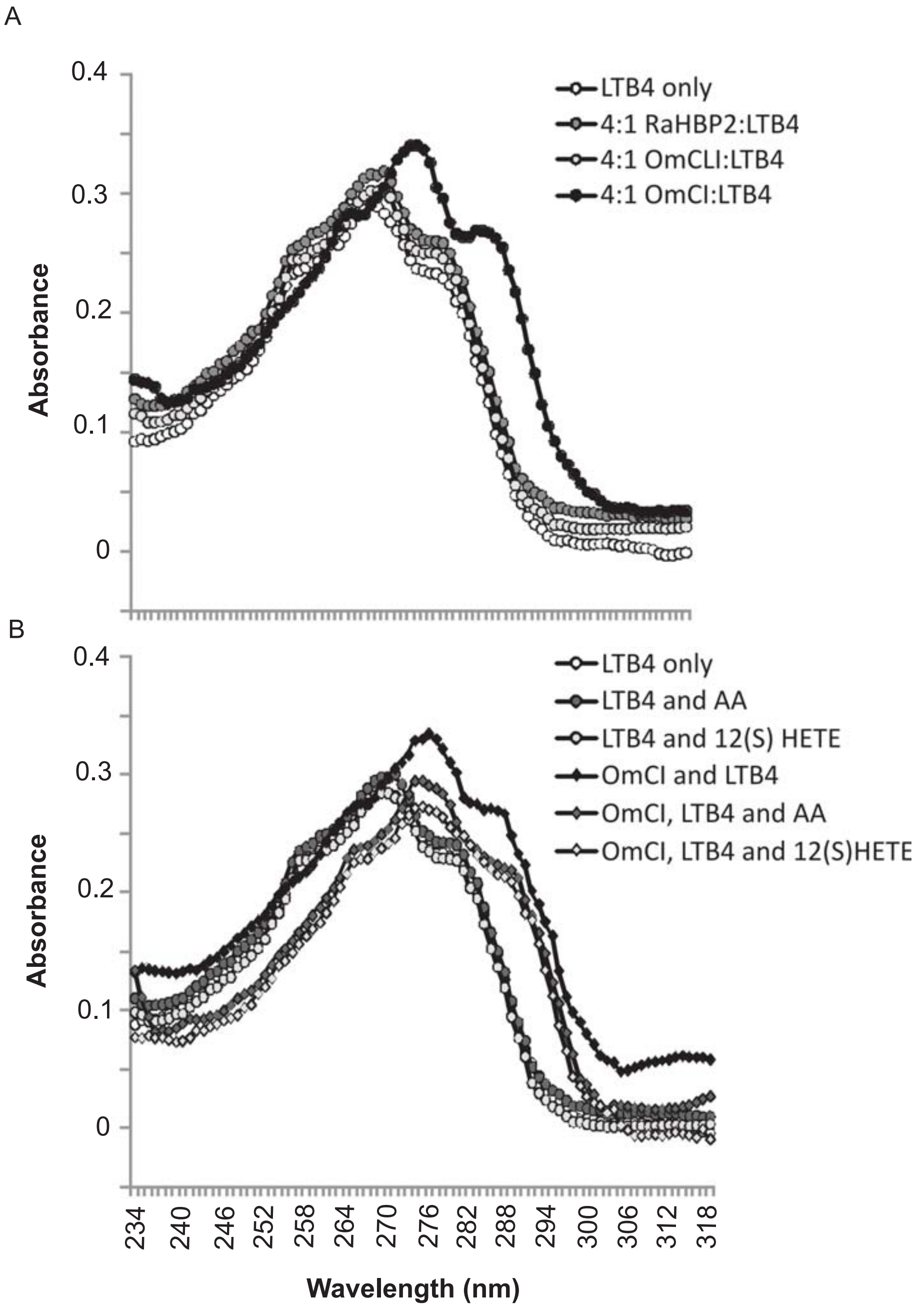
A
0.4
0.3
0.2
0.1
0
Absorbance
LTB4 only
4:1 RaHBP2:LTB4
4:1 OmCLI:LTB4
4:1 OmCI:LTB4
B
0.4
0.3
0.2
0.1
0
Absorbance
LTB4 only
LTB4 and AA
LTB4 and 12(S) HETE
OmCI and LTB4
OmCI, LTB4 and AA
OmCI, LTB4 and 12(S)HETE
234
240
246
252
258
264
270
276
282
288
294
300
306
312
318
Wavelength (nm)
