# Supplementary material for: Bifunctional Lipocalin Ameliorates Murine Immune Complex-induced Acute Lung Injury
Source: J Biol Chem. 2013 Apr 26;288(26):18789–802. doi: 10.1074/jbc.M112.420331 (PMC3696655; doi:10.1074/jbc.M112.420331)
Supplement: Supplemental Data [file supp_M112.420331_jbc.M112.420331-2.pdf]

Table SI. bOmCI data collection and processing

Dataset	bOmCI	bOmCI:LTB_4
X-ray source	ESRF ID14-2	ESRF BM14
Detector	ADSC	MAR CCD
Space Group (Z)	$P2_12_12_1$ (4)	$P2_1$ (8)
Unit cell parameters		
a (Å)	44.4	41.8
b (Å)	51.9	112.8
c (Å)	68.4	62.4
α (°)	90 º	90 º
β (°)	90 º	101.9º
γ (°)	90 º	90 º
Wavelength (Å)	0.93300	1.00384
Resolution Limits (Å)	68.4-1.71 (1.81-1.71)	113.0-1.86 (1.96-1.86)
Completeness (%)	89.8 (62.2)	96.9 (82.6)
Measured Reflexions	59386 (3426)	118155 (11352)
Unique Reflexions	15720 (1525)	46060 (5724)
Multiplicity	3.8 (2.2)	2.6 (2.0)
R_{merge}	0.035 (0.385)	0.033 (0. 495)
I/σ(I)	21.7 (2.0)	17.7 (2.1)

Values for the highest resolution shell are given in parentheses

Table SII. bOmCI structure refinements statistics

Crystal	bOmCI	bOmCI:LTB_4
Resolution range (Å)	38-1.71 (1.83-1.71)	50.0-1.86 (1.91-1.86)
Observations	15678 (1880)	45917 (2768)
Free set	783 (99)	2315 (135)
R(%)	0.198 (0.224)	0.220 (0.255)
Rwork (%)	0.197 (0.223)	0.220 (0.253)
Rfree (%)	0.223 (0.238)	0.238 (0.282)
Rmsd bond lengths (Å)	0.006	0.006
Rmsd bond angles (°)	0.93	0.87
Ramachandran outliers	0.0%	0.2%
Ramachandran favoured	97.2%	95.2%
Residues modelled (range)	145 (24-168)	4x145 (24-168)
Waters modelled	146	328
Average B (protein)	24.7	35.1
Average B (water)	37.6	37.0
Non-protein molecules	1xPalmitoleic Acid	4xLTB_4
PDB identifier	3ZUI	3ZUO

Values for the highest resolution shell are given in parentheses
